# Interaction of microtubules with the actin cytoskeleton via cross-talk of EB1-containing +TIPs and γ-actin in epithelial cells

**DOI:** 10.18632/oncotarget.12236

**Published:** 2016-09-24

**Authors:** Vera Dugina, Irina Alieva, Natalya Khromova, Igor Kireev, Peter W. Gunning, Pavel Kopnin

**Affiliations:** ^1^ Belozersky Institute of Physico-Chemical Biology, Lomonosov Moscow State University, Moscow, Russia; ^2^ Blokhin Russian Cancer Research Center, Moscow, Russia; ^3^ School of Medical Science, The University of New South Wales, NSW, Sydney, Australia

**Keywords:** EB1, +TIPs, microtubules, actin isoforms, β-actin, γ-actin

## Abstract

Actin microfilaments and microtubules are both highly dynamic cytoskeleton components implicated in a wide range of intracellular processes as well as cell-cell and cell-substrate interactions. The interactions of actin filaments with the microtubule system play an important role in the assembly and maintenance of 3D cell structure. Here we demonstrate that cytoplasmic actins are differentially distributed in relation to the microtubule system. LSM, 3D-SIM, proximity ligation assay (PLA) and co-immunoprecipitation methods applied in combination with selective depletion of β- or γ-cytoplasmic actins revealed a selective interaction between microtubules and γ-, but not β-cytoplasmic actin via the microtubule +TIPs protein EB1. EB1-positive comet distribution analysis and quantification have shown more effective microtubule growth in the absence of β-actin. Our data represent the first demonstration that microtubule +TIPs protein EB1 interacts mainly with γ-cytoplasmic actin in epithelial cells.

## INTRODUCTION

Actin microfilaments are highly dynamic cellular structures with their turnover time on the order of seconds at the cell periphery [1]; nevertheless, they effectively regulate cell shape. Microtubules are also highly dynamic - they are continuously growing or shortening even when no visual changes can be observed in the region of the cytoplasm they occupy. The ends of individual microtubules are growing or disassembling over distances of several microns [2, 3] and the whole system is continuously exchanging with the pool of monomeric tubulin with a turnover time of 5-20 min [4,5]. Microtubule assembly is characterized by enrichment of newly polymerized portions (plus ends) of microtubules with GTP- tubulin, and microtubule plus-end-tracking proteins (+TIPs) which specifically bind to these fragments [6, 7]. +TIPs represent a large group of structurally and functionally diverse microtubule regulators (End-Binding (EB) proteins; Cytoplasmic Linker Proteins (CLIP); CLIP Associated Proteins (CLASP) etc.), which are involved in microtubule interaction with the cell cortex [8]. The reciprocal interaction between microtubules and the actin cytoskeleton is necessary for important biological processes, including the establishment of cell shape and its maintenance, cell migration and division, intracellular transport and intercellular interactions [8, 9]. Previous studies suggested direct or indirect interaction between these two cytoskeletal systems. It was shown that microtubules can control the organization of the actin cytoskeleton by inducing local changes in the actomyosin contractility at the ends of stress-fibers [10]. Microtubule-actin interaction is the basis for endothelial cell barrier function [11-14].

The actin cytoskeleton in non-muscle cells is formed by two actin isoforms: non-muscle β- and γ-cytoplasmic actin (β- and γ-actin), encoded by *ACTB* and *ACTG1* genes respectively. They are ubiquitously expressed in cells [15, 16] and are essential for cell survival [17]. The b/γ actin ratio depends on the cell type [15, 18-20]. Modulation of actin isoform expression is often connected with different pathological processes [21] and gene transfection studies have shown that the two actin isoforms have opposing impact of myoblast architecture [22]. Previously, using specific monoclonal antibodies to β- and γ-actins and siRNA depletion of each cytoplasmic actin, we showed a preferential role for β-actin in contractile and adhesion structures; γ-actin has an important role in the formation of the cortical network necessary for cell shape flexibility and motile activity in normal fibroblasts and epithelial cells [23]. Both cytoplasmic actins were visualized at the apex of polarized epithelial cells in close proximity to intercellular contacts [23, 24], but these isoforms regulate different junctional complexes in epithelial cells. β-actin is connected to adhesion junctions, whereas γ-actin is connected to tight junctions [25]. Selective siRNA-mediated knock-down of γ-cytoplasmic actin, but not β-actin, induced epithelial to myofibroblast transition (EMyT) of different epithelial cells [26]. The EMyT manifested by increased expression of α-smooth muscle actin, and other contractile proteins, along with inhibition of genes responsible for cell proliferation. These findings demonstrated unique role of γ-actin in regulating epithelial phenotype and suppression of EMyT that may be essential for cell differentiation and tissue fibrosis [26].

These two actin isoforms play different roles in neoplastic cell transformation. Recently we have shown that β-cytoplasmic actin acts as a tumor suppressor, affecting epithelial differentiation, cell growth, cell invasion of colon and lung carcinoma cells *in vitro* and tumor growth *in vivo.* In contrast, γ-cytoplasmic actin enhances malignant features of tumor cells whose actin network regulation is carried out *via* the γ-actin isoform [27]. The goal of this study was to identify an actin isoform-specific interaction between microtubules and actin cytoskeleton.

## RESULTS

### Cytoplasmic actins are differentially distributed in relation to microtubule system in 3D cell architecture

**Figure 1:**
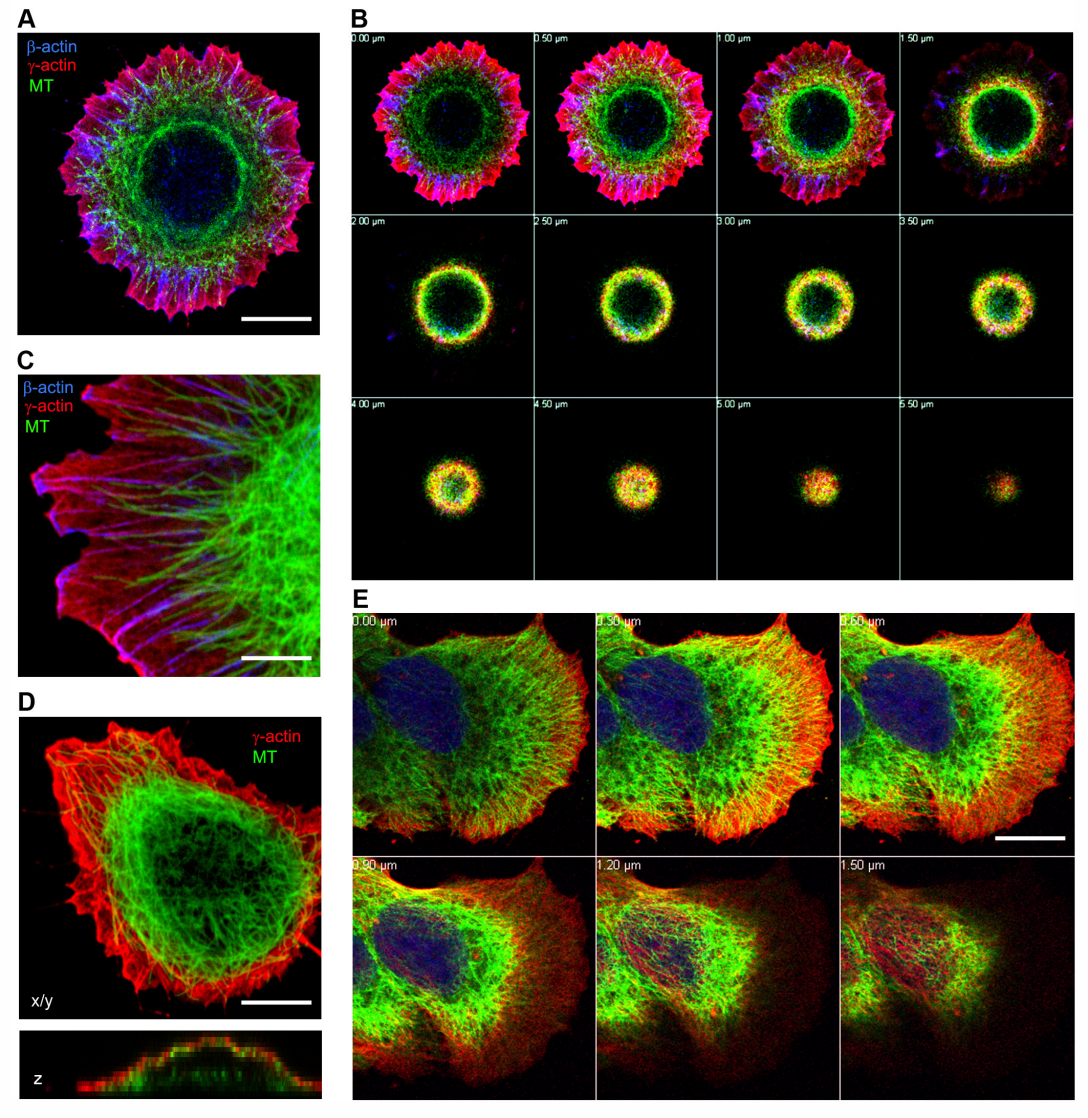
Subcellular localization of cytoplasmic actins and microtubules in spreading epithelial cells HaCaT **A.**-**D.** or MCF-7 (E) cells were plated for either 6 (A, B, C) or 16 hours (D, E) and stained for β-actin, γ-actin and α-tubulin. Images represent single X/Y sections (A, C, D) and Z section (D, bottom image). Panel B and E represent galleries of optical sections taken with 0.5 μm (B) or 0.3 μm **E.** step from the ventral (close to the substrate, first image) to the dorsal (last image) side of the HaCaT (B) cell shown in Fig 1A or MCF7 cell (E). Microtubules are distributed in close proximity to the γ-actin network, but not codistributed with the β-actin bundles. Bars, 5 μm (C) and 10 μm.

3D cell architecture depends on cell functions derived from interactions between actin filaments and the microtubule system. Two main layers of the actin filament system in the cell could be distinguished by super-resolution microscopy [28]: apical or dorsal and ventral. The apical (dorsal) organization of actin contains the cortical γ-actin microfilament network shown by LSM [23]. Previous studies have mainly visualized microtubules in 2D using TIRF microscopy [10, 29] and the cortical compartment of the cell was not detected by this method. We first addressed the location of the microtubule radial system in 3D, especially in spreading cells, where the difference between the actin isoforms is more obvious [23]. Confocal immunofluorescent microscopy verified that in spreading epithelial cells β-actin forms short bundles at the basal level and γ-actin is located in the cortical level and in the lamella (Figure 1A and 1B, Figure S1 A and B). Microtubules are distributed through all z-levels (Figure1B, optical z-sections), they are overlapped with γ-actin network, but they are not co-localized with β-actin structures in lamellae (Figure 1C, Figure S1C). The 3D interrelationship between the γ-actin cortical network and microtubules is evident in spreading HaCaT cells (Figure 1C and 1D), as well as in neoplastic MCF-7 cells (Figure 1E). Initial LSM visualization shows compartmentalization of β- and γ-actins (Figure 1), as well as close connection between the microtubule system and the γ-actin cortical network, compared with segregation between microtubules and the β-actin basal bundles (Figure 1A-1C). However, the resolution of the LSM along the z-axis does not allow us to distinguish the details of the superposition of both systems.

**Figure 2:**
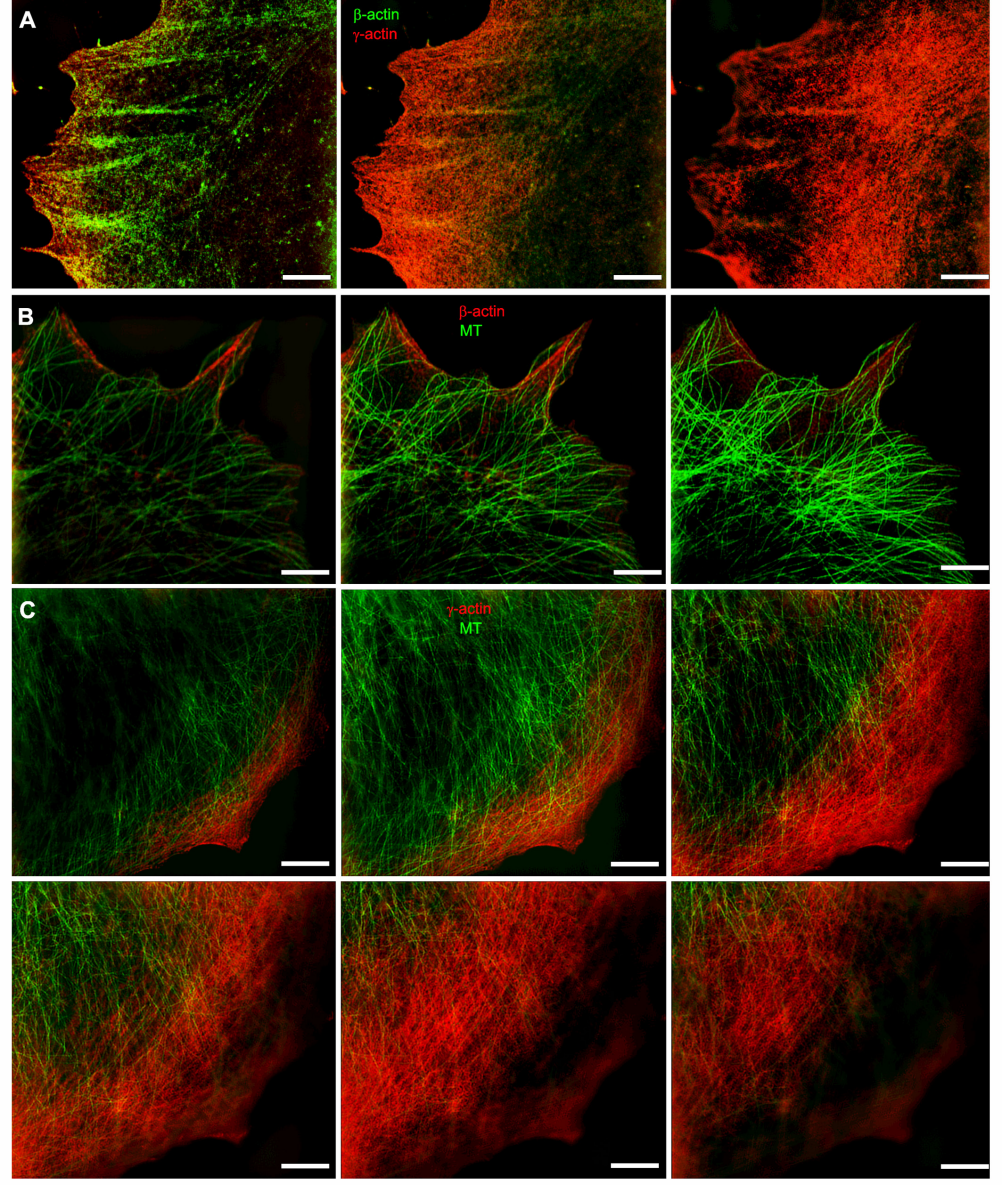
Codistribution of cytoplasmic actins with microtubules at the leading edge of MCF7 cells **A.** The β-actin bundles located at the basal level of the cell, closer to the substrate, and the cortical γ-actin network at the upper cell levels. **B.** Distribution of β-actin (basal level) and microtubules (upper level). **C.** Microtubules and γ-actin network codistribution. Microtubule tips are in close proximity to the γ-actin network. All panels represent galleries of optical sections taken with 0.12 μm step from the ventral (close to the substrate, first image) to the dorsal (last image) side of the lamella, SIM. Bars, 5 μm.

**Figure 3:**
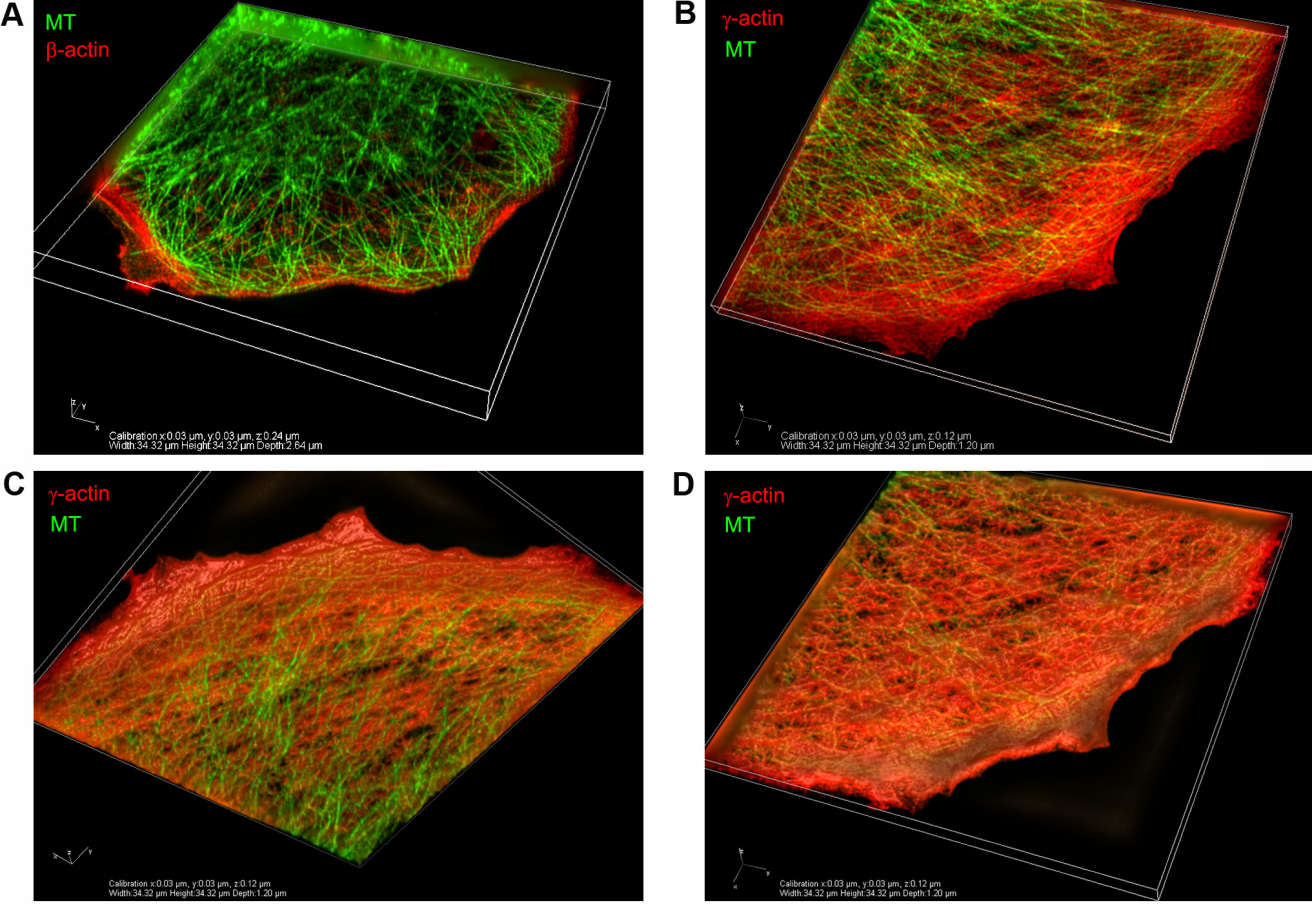
Codistribution of γ-actin with microtubules at the leading edge of MCF7 cells 3D-SIM. **A.** The β-actin basal bundles and the microtubule system, maximum intensity projection. **B.** The γ-actin cortical network and the microtubule system, maximum intensity projection. **C.**, **D.** The γ-actin cortical network and the microtubule system, average intensity projection. View from the bottom (C) or from the top (D) of the cell.

**Figure 5:**
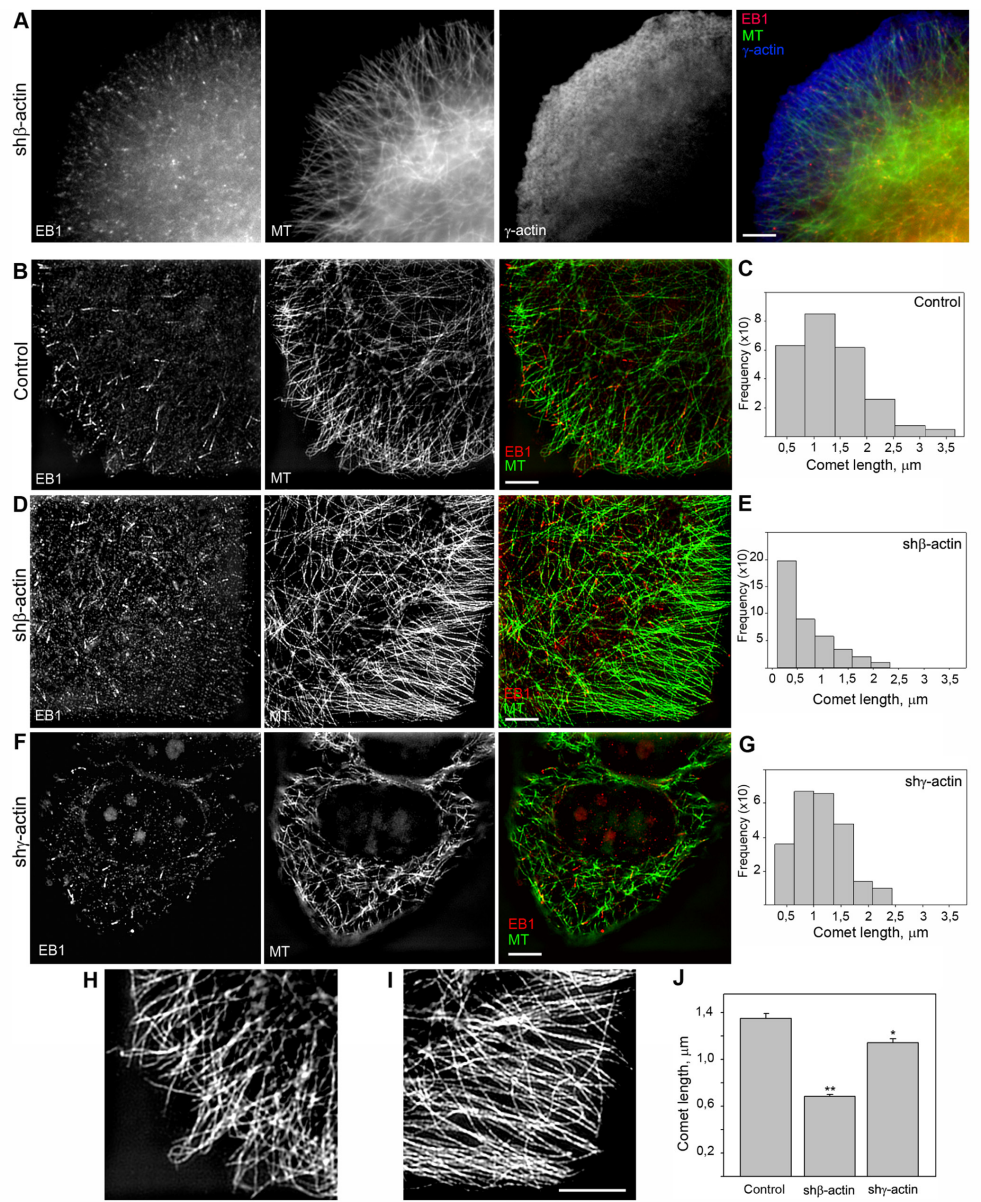
EB1-positive comet distribution and quantification **A.** EB1 is located at the terminal parts of radial microtubules. Triple IF staining of tubulin, EB1 and γ-actin, 3-color SIM/STORM imaging. **B.**, **D.**, **F.** Two-color SIM imaging of EB1 and γ-actin in control (B), shβ-actin (D) and shγ-actin cells (F). **C.**, **E.**, **G.**, **J.** Comet length quantification. **H.** Microtubule distribution at the leading edge of control (left) and shβ-actin (right) cells. Bars, 5 μm.

The relative positions of actin systems containing the two different cytoplasmic actin isoforms and the microtubules were studied using 3D-SIM super-resolution microscopy. Super-resolution SIM microscopy allowed us to detect several distinct layers of cytoskeletal structures along the z-axis: the cortical (dorsal) γ-actin network, basal β-actin filament bundles and the tubulin microtubule system between the two actin isoform layers (Figure 2A-2C). Microtubules run from dorsal layers beneath the γ-actin network towards the leading edges of the cell where their plus-ends terminate in close proximity to the short β-actin bundles (Figure 3A). Radial microtubules have different directionality and very often turn tangentially in control MCF7 cells (Figure 3A). The radial microtubule 3D-system is arranged in all volumes of the x/z axis with peripheral microtubules terminating near the γ-actin cortex (Figure 3B-3D). These 3D-SIM results allow us to discern clearly the separate distribution of non-muscle actin isoforms and to visualize that the radial microtubule 3D-system is filling the space between basal microfilament bundles and the cortical actin system. We investigated the pattern of interaction of the γ-actin cortex and the microtubule system using 3D-SIM microscopy (Figure 2C; Figure 3B-3D and Figure 5A). 3D-SIM microscopy showed the localization of the microtubule termini within the γ-actin cortical layers in each thin (0.12 μm) z-step.

**Figure 4:**
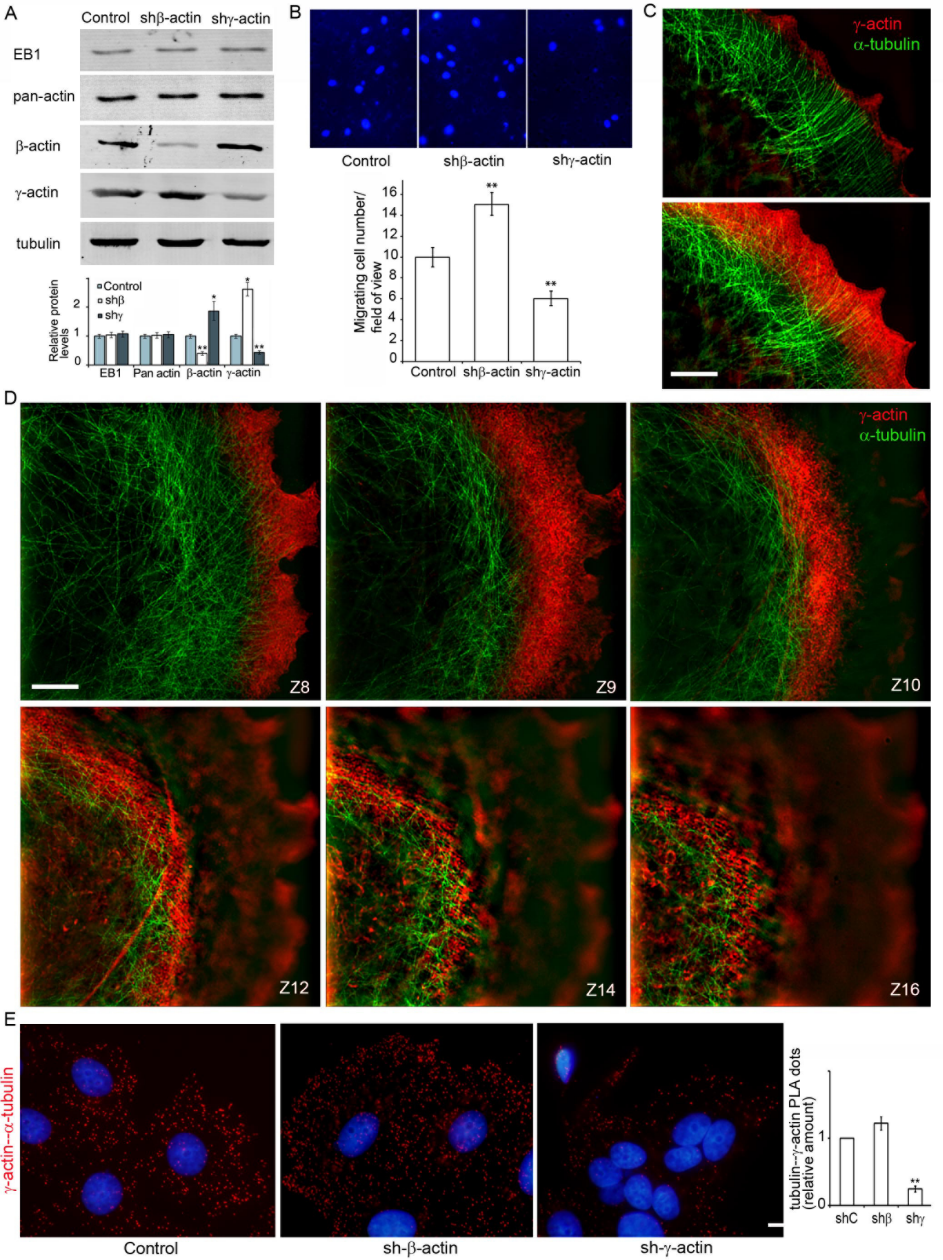
γ-actin-microtubule interaction after actin isoform down-regulation **A.** Down-regulation of cytoplasmic actins in MCF7 cells. WB analysis. **B.** Migratory activity of MCF7 cells with down-regulated β- or γ-actins. DAPI staining (upper panel) and quantification (Mean ± SEM; lower panel) of migrating cells. **C.**, **D.** Close distribution of microtubules and the γ-actin network at the leading edge (C, D) and in the cortex (D). All panels represent galleries of optical sections taken with 0.12 μm step from the ventral to the dorsal side of the lamella (C) or of the cell (D), SIM. Bars, 5 μm. E. γ-actin/α-tubulin PLA analysis of MCF7 cells with down-regulated β- or γ-actins. Graph represents relative amounts of PLA dots (Mean ± SEM). Bar 10 μm.

We generated MCF7 derivatives with depleted β- or γ-actins (Figure 4A) using shRNAs specific for each cytoplasmic actin isoform. The total amount of cytoplasmic actin remained unchanged indicating compensatory up-regulation of the alternative isoform. The MCF7 culture shows moderate levels of β-actin staining, (mainly in disorganized β-actin bundles) and γ-actin cortical staining. shRNA-mediated down-regulation of β-actin in these cells resulted in better spreading and increased cell motility while down-regulation of γ-actin induced a more epithelial phenotype (Figure S3). Overexpression of β-actin in the MCF7 cells inhibited spreading and motility leading to a “more normal” epithelial phenotype, γ-actin overexpression also induced a “more transformed” phenotype (Figure S3). In Boyden chamber assay mirroring cellular characteristics linked to directional 3D motility and malignant features cells with down-regulated γ-actin demonstrated lower motility compared with control. On the contrary, MCF7 cells with down-regulated β-actin migrated through membrane filters more effectively (Figure 4B) as consistent with our previous work [27].

Silencing of β-actin not only led to up-regulation of γ-actin, but also induced enhancement of cortical γ-actin staining (Figure 4C, 4D; Figure S2A). 3D-SIM revealed co-localization of the cortical γ-actin network with microtubules in control and β-actin-depleted cells. To detect the interaction between microtubules and γ-actin, the proximity ligation assay (PLA) method [30, 31] was employed in cells with actin isoform depleted by shRNA (Figure 4E).

The PLA assay verified the α-tubulin−γ-actin interaction (Figure 4E). To target possible interacting proteins we used pairs of antibodies to one of the cytoplasmic actin isoforms and α-tubulin. PLA using antibodies to γ-actin and α-tubulin demonstrated strong, highly specific signals as multiple cytoplasmic dots in control and β-actin-deficient cells (Figure 4E). The quantification of comparative fluorescent signals of α-tubulin−γ-actin PLA dots in control and actin-depleted MCF7 cells is shown in Figure 4D, (right). Staining for β-actin and α-tubulin resulted in fluorescent signals similar to those seen after staining with β-actin antibody alone (Figure S4). These experiments showed for the first time that microtubules interact with γ-actin but not with β-actin in neoplastic epithelial cells.

### Co-distribution of endogenous EB1 with cytoplasmic γ-actin isoform

**Figure 6:**
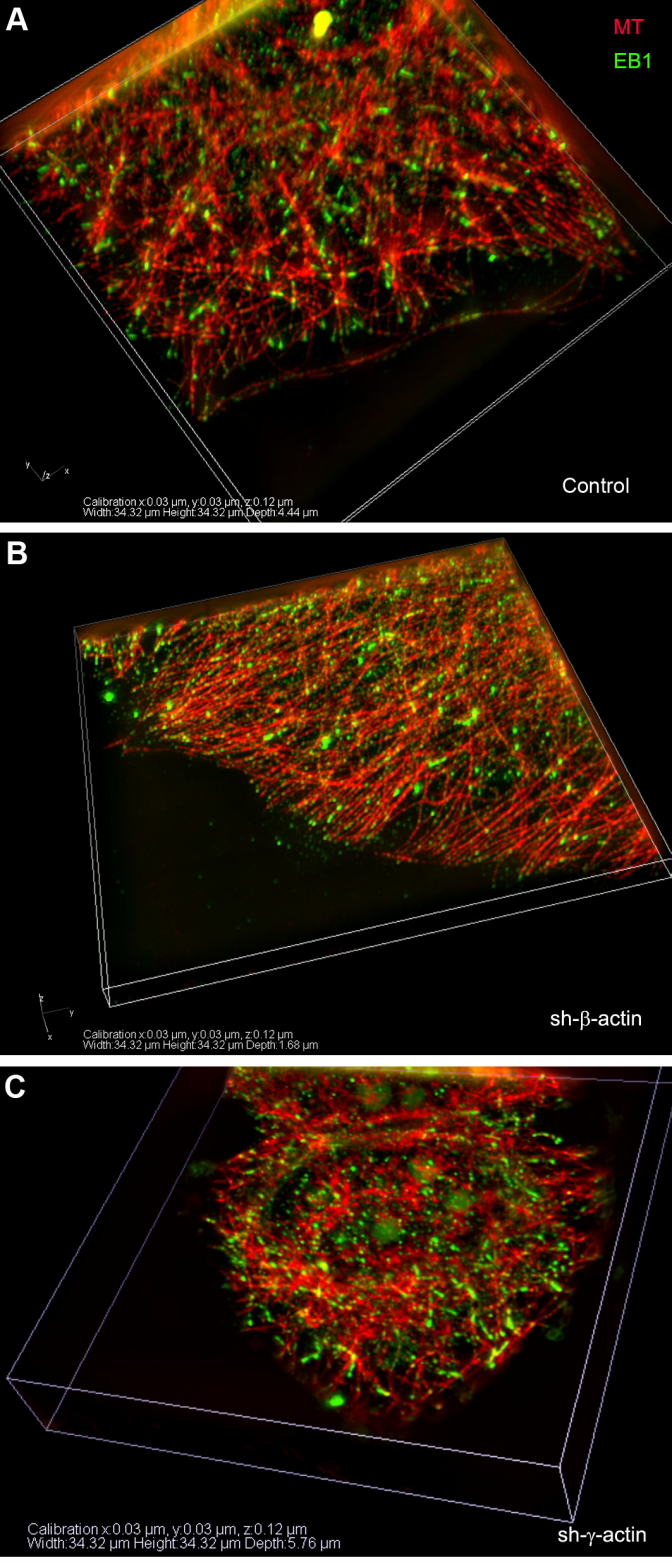
EB1-positive comet distribution in MCF7 cells with down-regulated β- or γ-actin 3D-SIM. A-C. Control, shβ-actin and shγ-actin cells.

The interrelationship between the cytoplasmic γ-actin isoform and the microtubule system was further studied using 3D-SIM microscopy in experiments with the microtubule +TIPs protein EB1. Microtubule plus-end tracking proteins (+TIPs) can be coupled to the actin cortical network through a protein complex including the +TIPs protein EB1 [32, 33]. EB1 puncta (”comet tails”) are located on the growing termini of microtubules and appear to be involved in microtubule dynamic instability [6]. In contrast to previous studies with exogenous expression of fluorescent +TIPs proteins, we performed immunofluorescent staining of endogenous EB1 in MCF-7 cells (Figure 5), to avoid the effect of over-expression. Two-color 3D-SIM imaging corroborated that EB1 is located at the terminal parts of radial microtubules (Figure 5B, 5D and 5F; Figure 6). The fraction of EB1 that co-localized with the terminal parts of radial microtubules was measured via the Manders' Overlap Coefficient [34]: 0,99±0,064 in control; 0,96±0,056 after β-actin depletion; 0,63±0,042 after γ-actin depletion. Triple IF staining of tubulin, EB1 and γ-actin using 3-color N-SIM imaging revealed many EB1 puncta localized in the γ-actin cortical network of MCF-7 cells (Figure 5A). 3D-SIM analysis of the cell leading edge after β-actin silencing also revealed that EB1 co-localized with well-developed γ-actin network (Figure 5) with the Manders' Overlap Coefficient 0,91±0,052 (0,86±0,045 in control). We did not use images of γ-actin depleted cells for co-localization analysis because of insufficient intensity of the fluorescence signal. The co-localization analysis of EB1 and γ-actin indicated that a microtubule - γ-actin cortex interaction may be mediated by the +TIPs protein.

### EB1-positive comet distribution and quantification

We used the microtubule plus end protein EB1 as the marker for selective visualization of the growing microtubule ends in control and β- or γ-actin depleted MCF7 cells. Quantitative analysis of the length and directionality of EB1-positive comets in lamellae of MCF7 cells was carried out using thin optical sections at basal cell level (0.240-0.360 μm from the substrate). Histograms of the length distribution of EB1 **с**omets were used for the comparative analysis (Figure 5С, 5E and 5G). The distributions in all experimental conditions were right-skewed using this class interval. The average comet's length in β-actin depleted cells was significantly shorter (0.68 ± 0.02 μm, *n* = 409, where n is number of events, 5 cells per group, 3 independent experiments) compared with control (1.35 ± 0.04 μm, *n* = 251) or γ-actin depleted cells (1.14 ± 0.03 μm, *n* = 241), indicating that microtubule dynamics is altered (Figure 5B-5G and 5J).

Microtubule organization was changed in the cytoplasm of β-actin- depleted cells as well as at the periphery (Figure 5B-5F; Figure S5, A). Microtubule reorganization was also observed at the leading edges of the cells with exogenous expression of β- or γ-actins (Figure S5, B). Microtubule density and fluorescence intensity at the leading edges of β-actin-depleted cells increased compared with control cells, whereas only integrated density value was significantly lower in γ-actin depleted cells compared with control (Figure S5, C and D). Microtubules were polymerized in a more straight manner, parallel to each other at the cell periphery (Figure 5H-5I) showing more effective growth in the absence of β-actin, whereas total tubulin polymerization level was increased in γ-actin-depleted cells (Figure S5, E). Histograms of the distribution of the angles between EB1 comets at the growing microtubule ends and radius vectors were used for the comparative analysis (Figure S6, A). The angles in β-actin depleted cells were homogeneous, indicating microtubule ends growing unidirectionally in these conditions only. The average angles between EB1 comets and radius vectors (Figure S6, B) were significantly decreased in β-actin depleted cells (5,6±0,8°) compared with control (38,6±3,6°); on the contrary, the average angles in γ-actin depleted cells were increased (50,6±4,1°).

### Selective interaction of endogenous EB1 with the γ-cytoplasmic actin isoform

**Figure 7:**
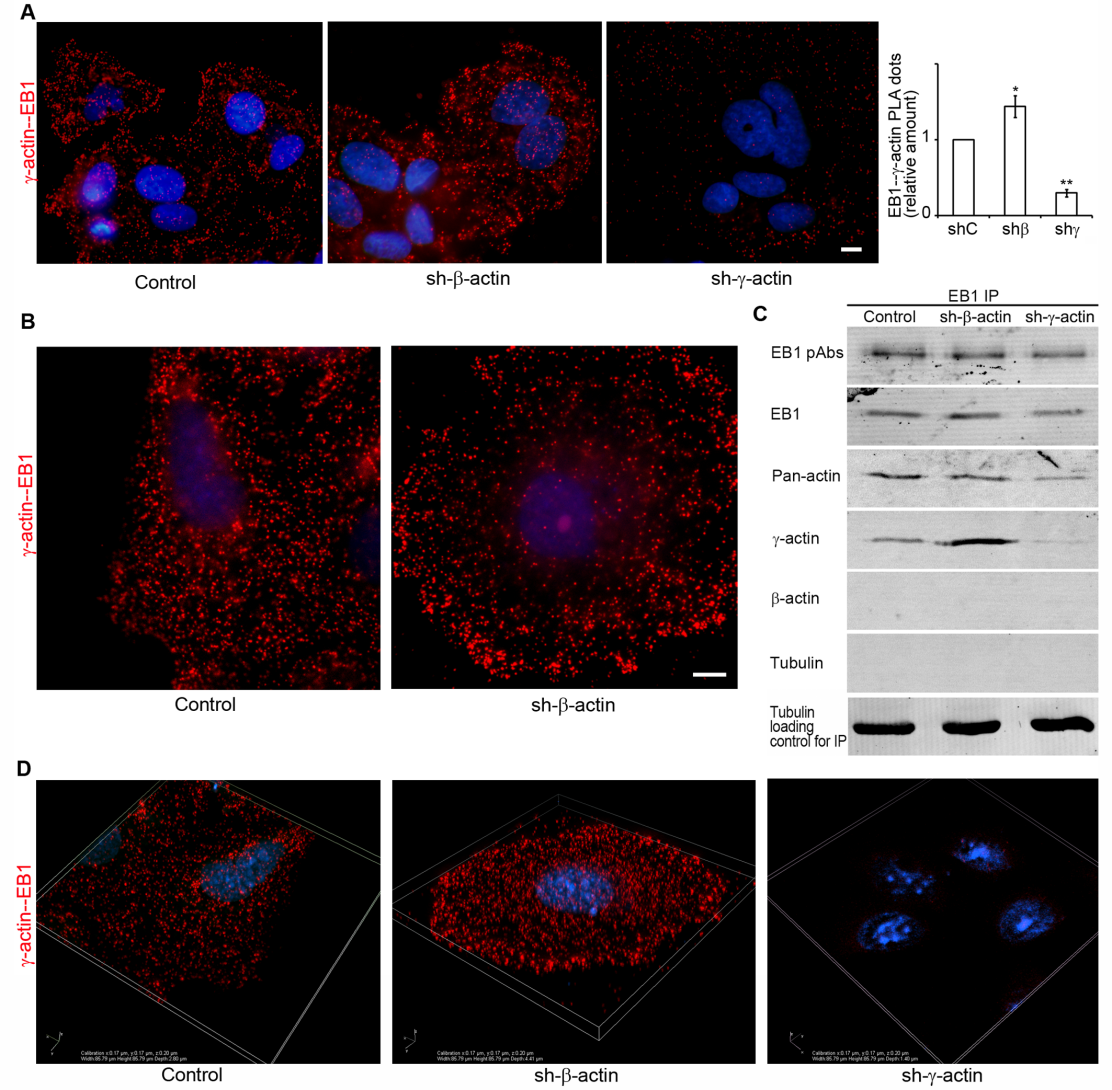
Selective interaction of EB1 with the cytoplasmic γ-actin isoform **A.** γ-actin/EB-1 PLA analysis of MCF7 cells with down-regulated β- or γ-actins. Graph represents relative amounts of PLA dots (Mean ± SEM). **B.** γ-actin/EB-1 PLA analysis of MCF7 cells, control cell (left) and cell with down-regulated β-actin (right), basal optical level after deconvolution. Bars, 10 μm. **C.** Co-immunoprecipitation (Co-IP) analysis with antibodies to EB1 after β- or γ-actin down-regulation. **D.** γ-actin/EB-1 PLA dots in MCF7 cells with down-regulated β- or γ-actins, 3D-SIM.

We performed *in situ* PLA to visualize EB1 interactions with actin isoforms in cell lines after γ- or β-actin depletion. PLA using antibodies to γ-actin and EB1 demonstrated strong, highly specific signals as multiple dots at the leading edge of control and, more obviously, of β-actin-deficient cells (Figure 7A, 7B and 7D). The quantification of comparative fluorescent signals of EB1−γ-actin PLA dots in control and actin-depleted MCF7 cells is shown in Figure 7A (right). PLA using antibodies to EB1 resulted in fluorescent signals similar to those seen after staining with β-actin antibody alone (Figure S4). 3D-IF microscopy (wide-field with deconvolution) of EB1−γ-actin PLA dots in control and actin-depleted MCF7 cells were performed. Different distribution in β-actin-deficient cells compared with control cells is shown (Figure 7B), where the difference in the pattern of PLA dots for γ-actin-deficient cells was more obvious. In addition to a quantitative difference between the mean value of PLA dots for control and β-actin-deficient cells, dots were generally distributed evenly in the cytoplasm of control cells, independently of the z-axis level. In contrast, non-homogenous distribution between different cell regions with an enrichment of PLA dots at the leading edge in β-actin-deficient cells was revealed (Figure 7D).

**Figure 8:**
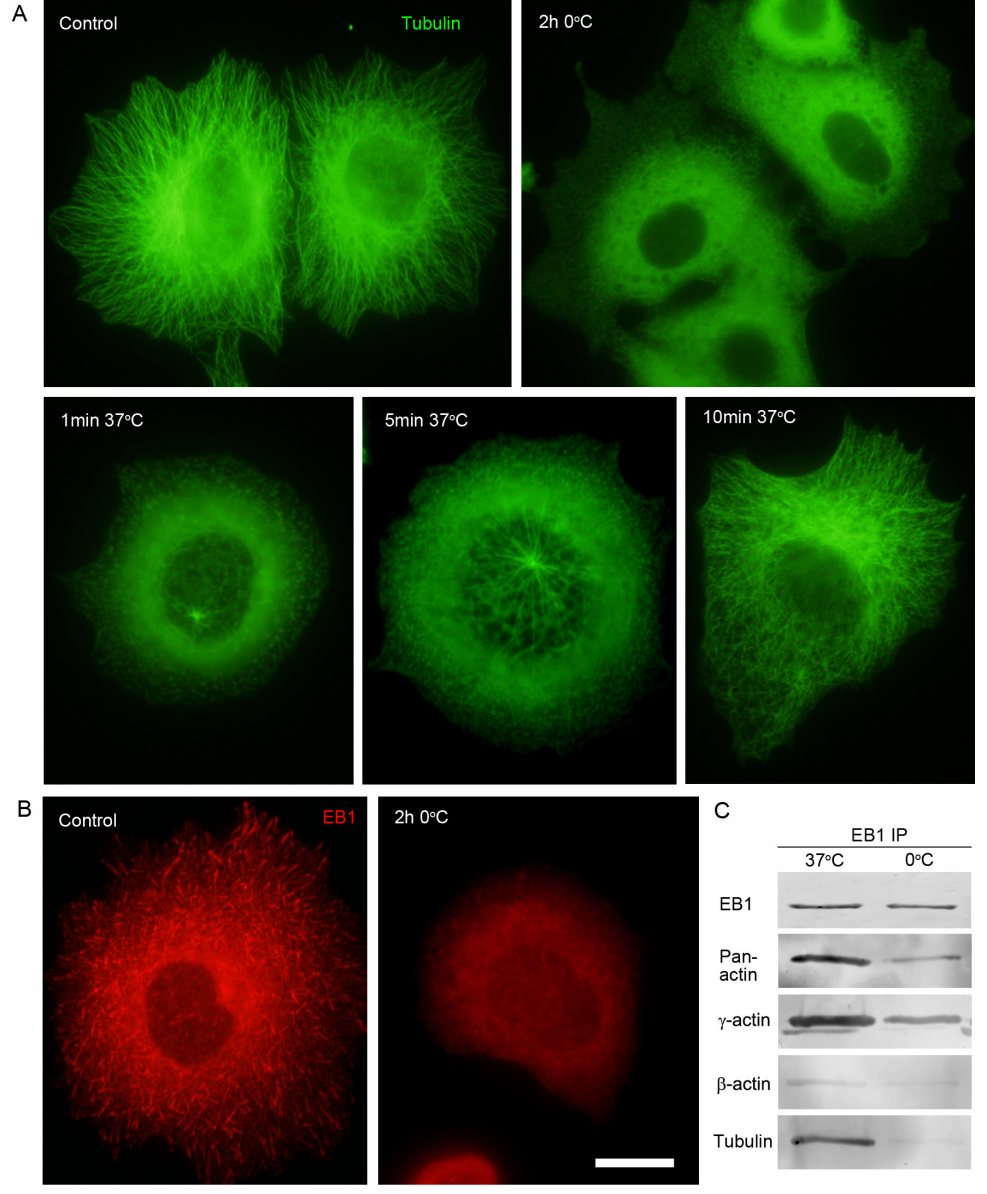
Interaction of EB1 with microtubules and the cytoplasmic γ-actin isoform in different conditions of tubulin polymerization **A.** Radial microtubule system at 37°C, diffuse tubulin after 2h incubation at O°C. Microtubule re-assembly in 1 min, 5 min and 10 min at 37°C after cold-induced de-polymerization. **B.** EB1 distribution pattern at 37°C and after 2h incubation at O°C. **C.** Co-IP analysis with antibodies to EB1 at 37°C and O°C. Bar 10 μm.

To reveal possible direct interactions of γ-actin with EB1 we used co-immunoprecipitation (Co-IP) analysis. First we checked the protein levels of cytoplasmic actin isoforms and EB1 after β- or γ-actin depletion (Figure 4A). No change of EB1 expression was detected after shRNA depletion of cytoplasmic actins in MCF-7 cells. Then Co-IP with antibodies to EB1 at 0°C was performed in cells depleted of β- or γ-actin. γ-Actin and EB1 were detected in the Co-IP with EB1-specific antibodies (Figure 7C), whereas β-actin and tubulin were not detected (Figure 7C). The absence of tubulin in this Co-IP analysis was not surprising, taking into account a well-known feature of EB1 to bind strongly to tubulin polymers, but only weakly to dimers (Ligon et al, 2006). We have searched for suitable conditions for microtubule re-assembly after cold-induced de-polymerization in MCF7 cells (Figure 8A and 8B) by immunofluorescence, in order that to make the Co-IP with EB-specific antibodies in ice-cold (at 0°C) and warm (at 37°C) conditions. γ-Actin and EB1 were detected in the Co-IP with EB1 at 0°C and 37°C (Figure 8C), whereas tubulin was not detected at 0°C. Tubulin was detected in Co-IP at a more physiological temperature of 37°C (Figure 8C).

**Figure 9:**
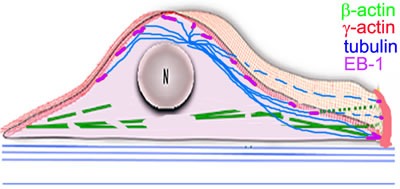
The radial 3D microtubule array in combination with the cytoplasmic actin Scheme

SIM and PLA analysis reveal co-localization of γ-actin and EB1 especially at the leading edge of lamella/lamellipodia. Co-IP experiments indicate that EB1 or a complex containing EB1 may interact with γ-actin, and microtubules are connected more to γ-actin, than to β-actin. Schematic presentation of our results with the radial 3D microtubule array in combination with the cytoplasmic actins is illustrated in Figure 9, where β- and γ-actins are segregated into dorsal and ventral compartments, respectively. This distribution is observed in all examined epithelial cell types, indicating that it represents a general feature.

## DISCUSSION

### Spatial segregation of microtubules and actin isoforms into different cell compartments

Using confocal immunofluorescence microscopy we previously discovered that the two cytoplasmic actins are segregated into dorsal and ventral zones in spreading normal keratinocytes and fibroblasts [23]. Moreover, these two actin isoforms are also segregated into different cell compartments in breast cancer cells in culture, in contrast to their homogenous membranous distribution in breast cancer lesions [24]. These two actin networks with distinct structural organization were also revealed by improved STORM with dual-objectives [28]. Two vertically separated actin layers were observed in the sheet-like cell protrusion despite its thinness. We propose that these two actin layers observed by STORM in BSC-1 epithelial cells, as well as in COS-7 fibroblast-like cells, correspond to β-actin ventral bundles and the γ-actin dorsal cortical network. Here we report that the radial microtubule system is localized between the two vertically separated layers of actin. The imaging capability of 3D-SIM did not allow us to observe individual filaments of cytoplasmic γ-actin in the cortical network, but the difference in localization of β- and γ-actin systems of microfilaments in relation to microtubules was observed. 3D microtubule arrays were located in close proximity to the cytoplasmic γ-actin cortical network.

### Microtubules may coordinate epithelial cell motility via EB1-γ- actin cross talk

The members of the growth-arrest-specific 2 (GAS2) family mediate the crosstalk between filamentous actin (F-actin) and microtubules, providing an essential link during axon extension [35-38]. Although all GAS2 proteins localize to actin and microtubules, only exogenously expressed G2L1 and G2L2 influence microtubule stability, dynamics and guidance along actin stress fibres [39]. This type of crosstalk between actin bundles and microtubules in fibroblasts is connected with cytoskeleton stability and maturation whereas in our experiments an interaction between microtubules and cytoplasmic γ-actin is more related to cell carcinoma cell motility. The coordination between the actin cytoskeleton and microtubules may be performed via guidance of microtubule growth by actin bundles [40]. This occurs in moving cells, where growing microtubule tips are targeted toward focal adhesions [29, 41-43]. The plus ends of dynamic microtubules can be coupled to the cell cortex via IQGAP1 [44] or APC [45].

EB1, the member of the +TIPs EB family, forms a comet-like accumulation and turns over rapidly at the microtubule plus end. Fluorescent microscopy revealed that up to a few hundred EB molecules bind to the region of growing microtubule ends, where they form comets that are 0.5-2.0 μm long [46-48]. EB1 comet length was changed in our experiments indicating that MT dynamics is altered by the actin isoform composition of the cell. In mammalian cells, EB1 has been shown to increase persistent microtubule growth and suppress catastrophe frequency [49]. *In vitro* studies have identified EB1 as a factor that decreases the maturation time of growing microtubule ends [50], connecting EB1 localization and regulation of MT dynamics. The length of EB comets reflects the size of the microtubule GTF cap [48]. The shortening of microtubule comets that we observed in β-actin depleted cells indeed usually correlates with frequent switching between very short periods of growth and shortening. In β-actin depleted cells microtubules are more parallel than in untreated cells; while in γ-actin depleted cells microtubules tend to become more entangled.

The important functional role of EB1 in cell motility was demonstrated in melanoma cells [51], where EB1 depletion decreased lamellipodia protrusion. Furthermore, EB1 overexpression correlated with glioblastoma progression and migratory potential, whereas downregulation of EB1 by shRNA inhibited cell migration and proliferation *in vitro* [52]. Modulation of γ-actin expression leads to similar functional changes in normal [53] and neoplastic cells [23, 27, 54]. We confirm here in Boyden chambers assay, that actin isoforms regulation leads to changes in migratory characteristics of carcinoma cells. Cells with down-regulated γ-actin demonstrated lower motility compared with control. On the contrary, MCF7 cells with β-actin depletion migrated more effectively.

The microtubule +TIPs protein EB1 is expressed at high level in breast cancer cells [55], which allowed us to readily detect the location of endogenous EB1 and the length of comet tips. In our experiments we observed that EB1 comet length was significantly changed in β-actin depleted cells. We and others have previously shown that EB1 comets become shorter after the treatment with low doses of microtubule-destabilizing drugs [7, 13] when microtubule growth rate is reduced. The shortening of microtubule comets predominantly near the cell edge of β-actin depleted cells correlated with microtubule reorganization at the periphery and suggests that the relative levels of the actin isoforms regulate microtubule dynamics. Moreover, microtubules were more Triton-X-100-resistant in γ-actin-depleted cells in our experiments with detergent fractionation of MCF7 cells. Higher stability of microtubules after γ-actin siRNA treatment have also been demonstrated in neuroblastoma cells [56]. Taken together, this data indicate that β- and γ-actin filament networks have an opposite impact on microtubule dynamics and organization.

Previous studies have suggested that +TIPs proteins may indirectly interact or mediate cross-talk between microtubules and the actin cytoskeleton [40, 43, 57]. Through a combination of biochemical and biophysical techniques the Goodson lab have found that EB1 can bind skeletal F-actin *in vitro* and that the F-actin binding site on EB1 overlaps with the well-characterized EB1-microtubule binding site. Competition experiments and mutagenesis data suggest that EB1-F-actin binding and EB1-microtubule binding are mutually exclusive. They suppose that this interaction may assist cells in differentially regulating microtubule stability in the actin-rich cortex as opposed to the cell interior [58]. In consistent with their data we have shown the possibility of microtubule-actin cortical network interaction in carcinoma cells. The interaction was prominent in the absence of β-actin bundles as opposed to bundle enrichment after γ-actin depletion.

We hypothesized that microtubules can interact with the γ-actin cortical network directly via a +TIPs protein complex. Our results obtained through super-resolution microscopy with colocalization analysis, PLA and CoIP all indicate that microtubules directly or indirectly bind to γ-actin via a +TIPs protein EB1-containing complex. Such a complex may include IQGAP, APC, Rac etc. Our recent paper revealed multiple interaction partners of γ-actin in lung and colon cancer cells [27], such as ERK1/2, p34-Arc, WAVE2, cofilin1, PP1. These proteins are involved in structural and signaling regulation of cell motility and proliferation. To our knowledge these data represent the first demonstration that the endogenous +TIPs protein EB1 interacts with one of the cytoplasmic actin isoforms. In agreement with our finding, recent data on neuroblastoma cells in mitosis [59] indicate that γ-cytoplasmic actin may regulate the microtubule apparatus of cancer cells.

### What is the biological significance of the microtubule-γ-actin interaction?

The “Search-and-Capture” model of microtubule dynamics [60] suggests that during transitions between growth and shortening of the plus ends microtubules rapidly explore the three dimensional intracellular space and search for targets to interact with or capture. We propose that a key target for microtubule exploration is γ-actin-containing filaments in the cell cortex. This provides a mechanism to link actin driven-remodeling of the cell cortex with microtubule dynamics. The precise functional implications of this interaction are not known but it is almost certain to provide the mechanism by which microtubules participate in the regulation of cell motility.

## MATERIALS AND METHODS

### Cells

Human breast adenocarcinoma MCF7 cell line (ATCC^®^ HTB22™) and human immortalized keratinocytes HaCaT (CLS # 300493) were used. pLKO.1 lentiviral DNA constructs together with the pΔR8.2 (#12263, Addgene) and pVSV-G (#8454, Addgene) packaging plasmids were transfected into 293FT packaging cells (R70007, ThermoFisher) using TurboFect Transfection Reagent (R0531, Thermo Scientific). Virus-containing supernatants were collected 24 to 48 h after transfection and used to infect the recipient cells in the presence of 8 μg/mL polybrene (SIGMA). Infected cell cultures were selected for 4-5 days in medium containing 1 μg/mL puromicin (SIGMA) for pLKO.1-puro constructs. All experiments were performed 5-8 days after vector-mediated gene transfer.

### DNA constructs

Previously selected and verified [27] for MCF7 and HaCaT cells β- and γ-actins specific siRNA-expressing constructs were used. Briefly, for β- and γ- actins si-dependent repression 21 nt target sequences 5′-CAAATATGAGATGCGTTGTTA-3′ corresponding to 1465-1475 of β-actin mRNA ref|NM_001101.3| and 5′-CAGCAACACGTCATTGTGTAA-3′ corresponding to 2057-2077 of γ-actin mRNA ref|NM_001199954.1 included in hairpin structures were cloned into pLKO.1 (SIGMA) lentiviral vector. pLKO.1-shGFP-puro targeting eGFP (GenBank Accession No.pEGFP U55761) was used as a control. To prove of β- and γ- actins mRNAs repression caused by si-RNA expression, total mRNA was isolated with SV Total RNA Isolation System (Promega) according to the manufacturer's protocols and mRNA amounts of β- and γ-actin by routine PCR were tested. The following primers were used: β-actin forward 5′-ACAGAGCCTCGCCTTTGC-3′, reverse 5′­GAGGCGTACAGGGATAGCAC-3′; γ-actin forward 5′-CAAAAGGCGGGGTCGCAA-3′, reverse 5′-TGGGGTACTTCAGGGTCAGG-3′; α-tubulin forward 5′­GTTGGTCTGGAATTCTGTCAG-3′, reverse 5′-AAGAAGTCCAAGCTGGAGTTC-3′. The quantification of mRNA bands was performed using Chemi-Smart 3000 Imaging System (Vilber Lourmat) and TotalLab v.2.01 software (data not shown). Oligonucleotides synthesis and DNA sequencing was performed by Evrogen (www.evrogen.com). Further, actin isoforms specific repression was tested by western-blot analysis. For β- and γ-actins overexpression human total cDNA's obtained from normal human fibroblasts was used for reverse PCR (oligo dT and M-MLV RT (Promega)) with specific β- and γ-actin primers with BamHI/EcoRI adapters (forward BamHI-β-actin 5′-ATATGGATCCATGGATGATGATATCGCCGCG-3′; forward BamHI-γ-actin 5′-ATATGGATCC ATGGAAGAAGAGATCGCCGCG-3′ and reverse EcoRI-β/γ-actin 5′- ATATGAA TTCCTAGAAGCATTTGCGGTGGACGAT-3′) and PfuUltraII (Stratagene) PCR products were cloned into the lentiviral pLenti6 vector (Invitrogen) with modified polylinker. Accuracy of PCR clones was verified by DNA sequencing.

### Western blot analysis

Whole cell extracts were lysed in ice-cold RIPA buffer (50 mM Tris-HCl pH 7.4, 150 mM NaCl, 1% deoxycholate Na, 1% NP-40, 0.1% SDS, 100 mM PMSF, 1 mM pepstatin A and 1 mM E64). Protein concentration in the extracts was determined with a protein assay system (BioRad). 5-20 μg of protein was separated on 8-12% SDS polyacrylamide gel and transferred to PVDF membrane (IPFL00010, Millipore). The membranes were blocked with SuperBlock Blocking Buffer (Thermo Scientific) and then probed with antibodies specific to corresponding proteins. Membranes were treated with Alexa488-conjugated secondary antibodies (A11029, Invitrogen), band detection was performed using variable mode imager Typhoon9410 (GE Healthcare).

### Detergent extraction

Equal amounts of cells were washed with HBSS buffer containing MgCl_2_ and CaCl_2_. Then cells were covered with minimal volume of HBSS buffer containing 0,05% or 0,02% detergent Triton-X-100 for 1 min with gentle shaking. Supernatants were collected and diluted with 2xGLB buffer. Residuary cells also were lysed in GLB buffer. Tubulin detection in obtained lysates was analyzed using western blot analysis.

### Immunoprecipitation

For co-immunoprecipitation assay cells washed with PBS were lysed at 0°C or 37°C in Pierce IP buffer (25mM Tris-HCl pH7.4, 150mM NaCl, 1mM EDTA, 1% NP-40, 5% glycerol) with proteases and phosphatases inhibitors (Roche). Lysates were cleared by 10 min centrifugation at 12000g. Overnight precipitation was performed with 2μg of EB1 antibodies, 500 μg of cell lysates pre-cleaned with normal horse serum and 50 μl of 50% Protein a/g Agarose (Invitrogen). Agarose beads were washed 5 times with IP buffer with centrifugation at 2000g and boiled in SDS-loading buffer. Not precipitated lysate aliquots were used as loading control.

### Antibodies

Mouse monoclonal antibodies to: β-actin (MCA5775GA, AbD Serotec); γ-actin (MCA5776GA, AbD Serotec); pan actin (4968, Cell Signaling); α-tubulin (2144, Cell Signaling); rat monoclonal antibodies to α-tubulin (Serotec); rabbit polyclonal antibodies to EB1 (Gen Script) and tubulin (Sigma-Aldrich).

The following secondary antibodies were used: AlexaFluor488-, AlexaFluor594-, AlexaFluor647-, Cy5-conjugated goat anti-mouse IgG1, IgG2b, IgG, goat anti-rabbit IgG and goat anti-rat IgG (Southern Biotechnology, Associates Inc., Birmingham, AL; Jackson; Life technologies).

### *In situ* proximity ligation assay (PLA)

The assay was conducted according to manufacturer's instructions (Sigma-Aldrich). In brief, PFA and methanol-fixed cells were incubated with pairs of primary antibodies (e.g. EB1/γ-actin, EB1/β-actin, EB1/pan actin), washed and incubated with secondary antibodies conjugated with oligonucleotides (PLA probe MINUS and PLA probe PLUS). A proximity-dependent ligation and synthesis of an amplifiable DNA reporter molecule followed. Amplification of the reporter molecule was established via rolling circle amplification and the resulting rolling circle products (dots) were visualized by hybridization with a complementary fluorescence-labeled oligonucleotide probe. The signal was visualized with fluorescent microscopy.

### Immunofluorescent and Confocal Laser Scanning microscopy

Cells were grown on coverslips, rinsed with pre-warmed DMEM containing 20mM Hepes at 37°C, fixed in 1% PFA in DMEM/Hepes for 10 min and treated for 5 min with MeOH at −20°C.

For EB1 and tubulin detection fixation with MeOH at −20°C was used. Cells were incubated with primary and secondary antibodies. DAPI (Life technologies) was applied for nuclear staining.

Confocal images were acquired using laser scanning confocal microscope with 405 nm 5mW, 488nm 10mW, 555nm 10 mW, 639 nm 5 mW lasers (LSM510, Zeiss, Oberkochen, Germany) equipped with oil Plan-Fluar immersion objective, 100x /1.45 (Zeiss). Single optical sections were scanned with ~1μm thickness near the basal level of the cell. For serial optical sections stacks with Z-step of 0.2-0.5 μm were collected.

### 3D-SIM

Samples were mounted in Mowiol 4-88 (Calbiochem) with 1% DABCO to prevent bleaching and examined using a Nikon N-SIM (Nikon) with 100x/1.49 NA oil immersion objective, 488 nm and 561 nm diode laser excitation. Image stacks (z-steps of 0.12μm) were acquired with EMCCD camera (iXon 897, Andor, effective pixel size 60 nm). Exposure conditions were adjusted to get typical yield about 5000 max counts (16-bit raw image) while keeping bleaching minimal. Serial optical sections of the same cell taken in wide-field mode were deconvolved using the AutoQuant blind deconvolution algorithm. Image acquisition, SIM image reconstruction and data alignment were preformed using NIS-Elements 4.2 software (Nikon).

### EB1-positive comet guantitative analysis

We used MT plus end protein EB1 as the marker for selective visualization of growing MT ends in control and β- or γ-actin depleted human breast adenocarcinoma MCF7 cells. Analysis of EB1-positive comets of growing microtubule plus ends was performed on immunofluorescent images prepared with antibodies to α-tubulin and EB1. Images were acquired using an N-SIM super-resolution system (see above). Original images were processed using ImageJ software (Gaussian filtration and background subtraction).

Quantitative analysis of the length of EB1-positive comets in lamellas of MCF7 cells was carried out using thin optical sections at basal cell level (0,240-0,360 μm from the substrate). Measurements of the length of EB1 сomets were produced using ImageJ software. Statistical analysis was performed using Sigma Plot 13.0 (SPSS Science, Point Richmond, CA) and Microsoft Excel. To describe microtubule system organization near the cell margin we used SIM images of cells stained for EB1. Relative positions of growing microtubule plus end comets were analyzed. We measured the angle between the vector, which the growing comet of microtubule plus end tip forms and the radius vector connecting the geometrical cell center with the cell periphery at the head of the comet. Statistical analysis was carried out using ImageJ and Sigma Plot 13.0. Sigma Plot 13.0 was used for graphical data presentation. Statistical analysis was performed using Student's t test; *p ≤ 0.05; **p ≤ 0.01. Error bars represent SEMs, except where noted.

### Boyden chamber cell migration assay

was performed using transwell chambers with 8-μm pore-size membranes (BD Biosciences) according to manufacturer instructions with 5×10^4^ MCF7 cells from 2 to 20% of FCS. The migration activity was quantified by blind counting of the migrated cells from 20 fields per chamber in 3 independent experiments (Mean ± SEM).

### Statistical analysis

Data obtained were analyzed statistically using ImageJ, Sigma Plot 13.0 and Excel software. Statistical analysis was done with unpaired Student's *t* tests, and data are expressed as Mean ± SEM as indicated in figure legends. *P* values (*) ≤ 0.05 and (**) ≤ 0.01 were considered to be significant. All the experiments were performed for at least three times.

## SUPPLEMENTARY MATERIALS FIGURES


