# Supplementary material for: Interaction of microtubules with the actin cytoskeleton via cross-talk of EB1-containing +TIPs and γ-actin in epithelial cells
Source: Oncotarget. 2016 Sep 24;7(45):72699–715. doi: 10.18632/oncotarget.12236 (PMC5341938; doi:10.18632/oncotarget.12236)
Supplement: Supplementary file 1 [file oncotarget-07-72699-s001.pdf]

Interaction of microtubules with the actin cytoskeleton via cross-talk of EB1-containing +TIPs and γ-actin in epithelial cells

Supplementary Material

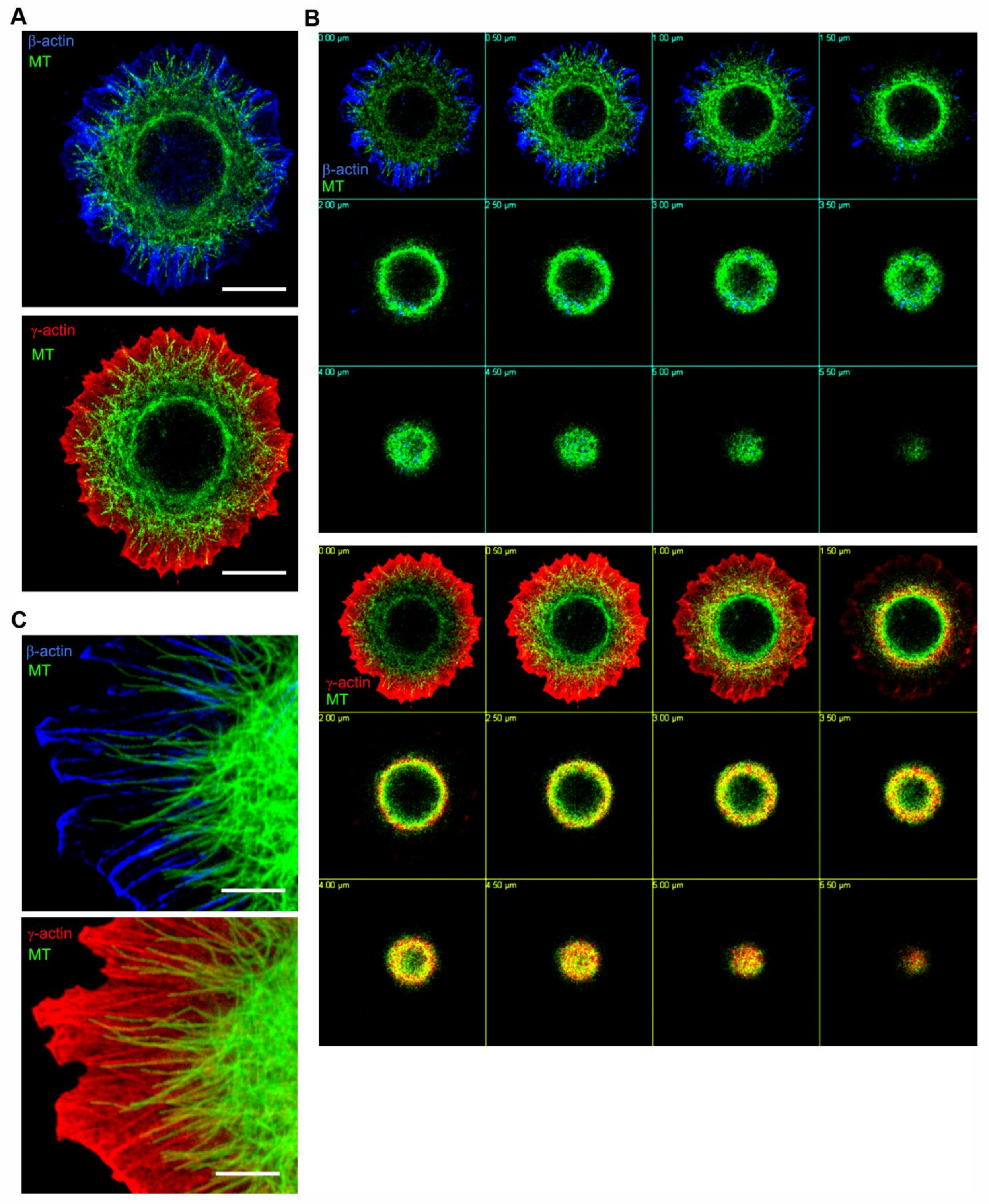

Supplementary Figure 1. Subcellular localization of cytoplasmic actins and microtubules in spreading epithelial cells (triple staining separated into paired channels).

A-C. HaCaT cells were plated for 6 hours and stained for β-actin, γ-actin and α-tubulin. Images represent single X/Y sections (A, C). Panels B represent galleries of optical sections taken with 0.5 µm step from the ventral (close to the substrate, first image) to the dorsal (last image) side of the HaCaT cell shown in Fig.1A. Microtubules are distributed in close proximity to the γ-actin network, but not codistributed with the β-actin bundles. Bars, 10 µm (A) and 5 µm (C).

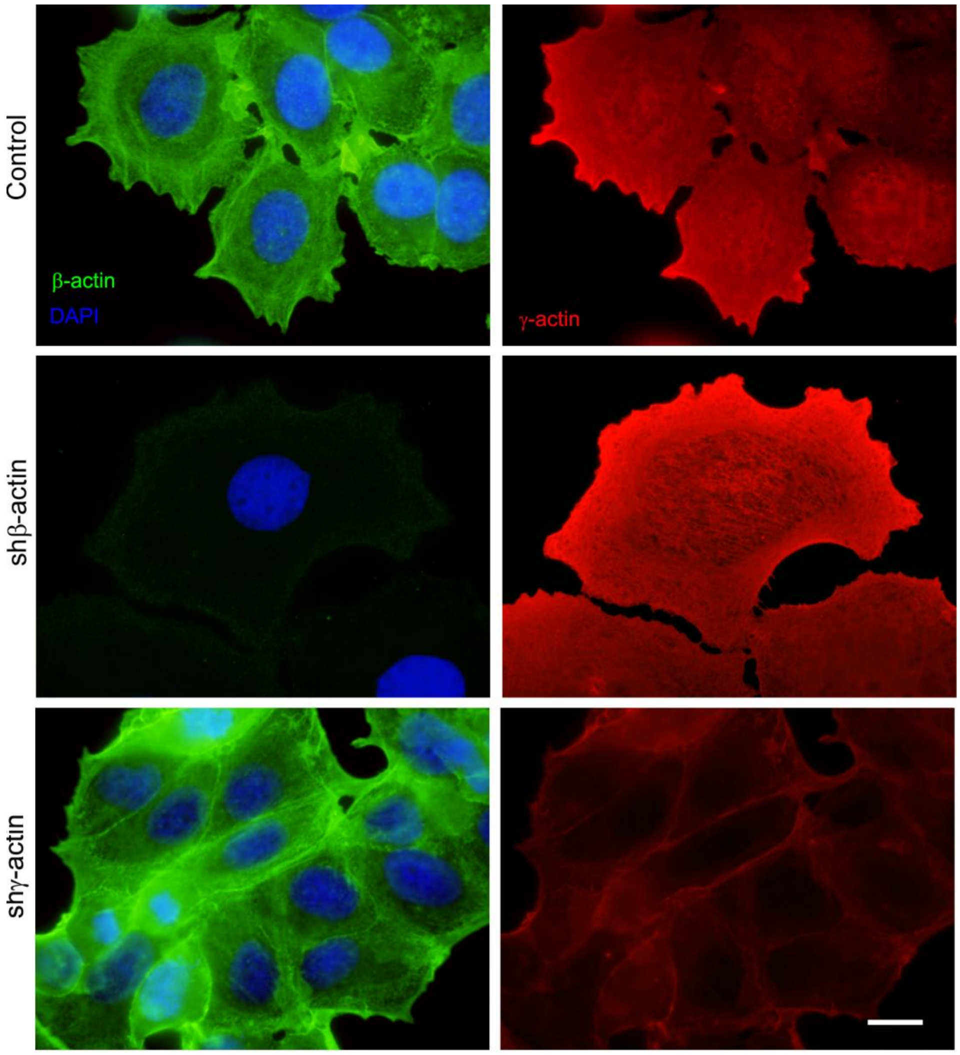

Supplementary Figure 2. Downregulation of β- or γ-actin expression by corresponding shRNAs in tumor MCF7 cells leads to phenotypical changes.

Immunofluorescent staining of MCF7 cells with β- or γ-actin depletion by corresponding shRNAs. Both β- and γ-actin depletion induce major morphological changes. Bar, 10µm.

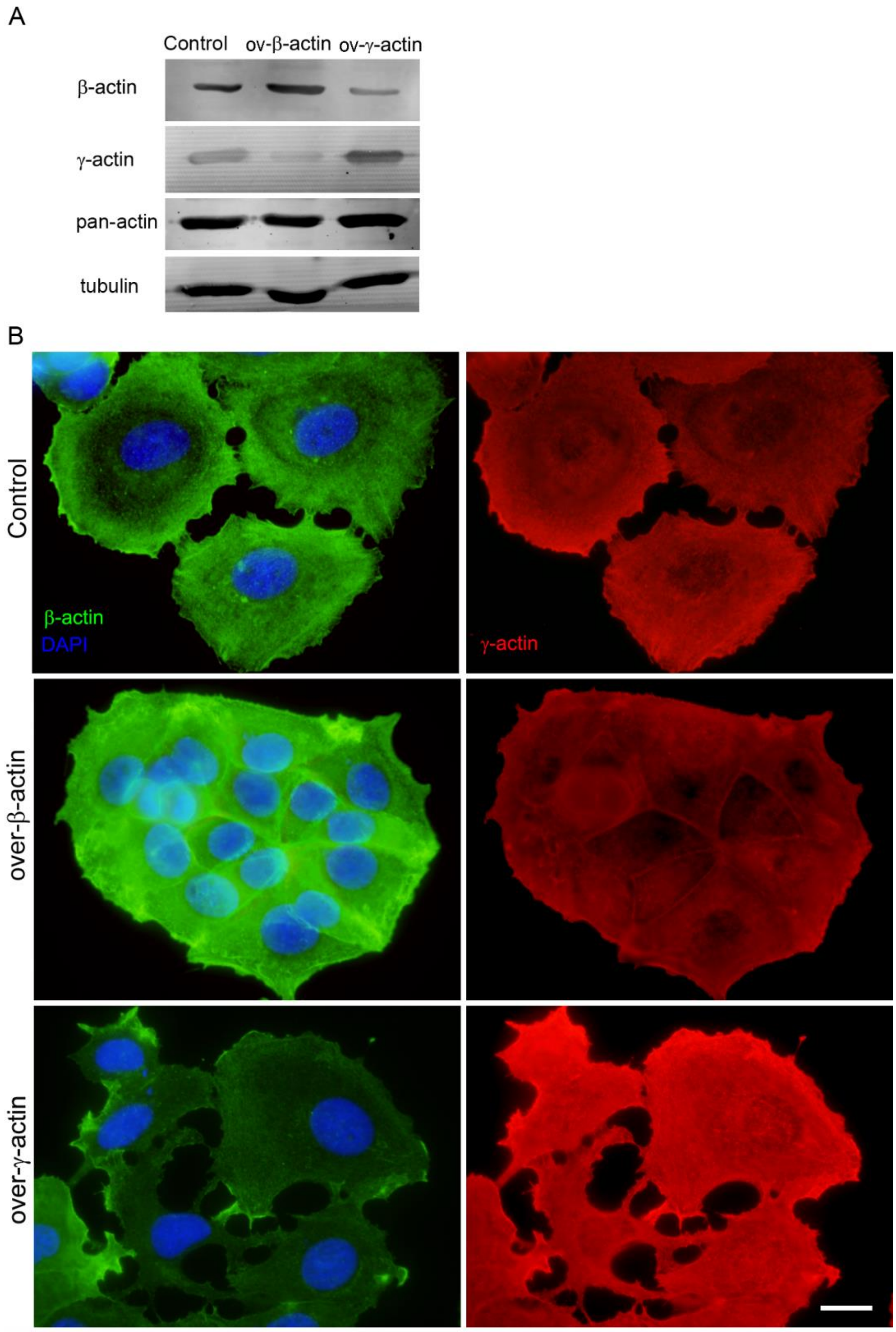

Supplementary Figure 3. Exogenous expression of β- or γ-actin in tumor MCF7 cells leads to phenotypical changes.

A. WB analysis of MCF7 cells with exogenous expression of β- or γ-actins.

B. Immunofluorescent staining of MCF7 cells with exogenous expression of β- or γ-actin. Both β- and γ-actin overexpression induce major morphological changes. Exogenous expression of β- or γ-actins leads to phenotypes similar to γ- or β- actin depletion. Bar, 10μm.

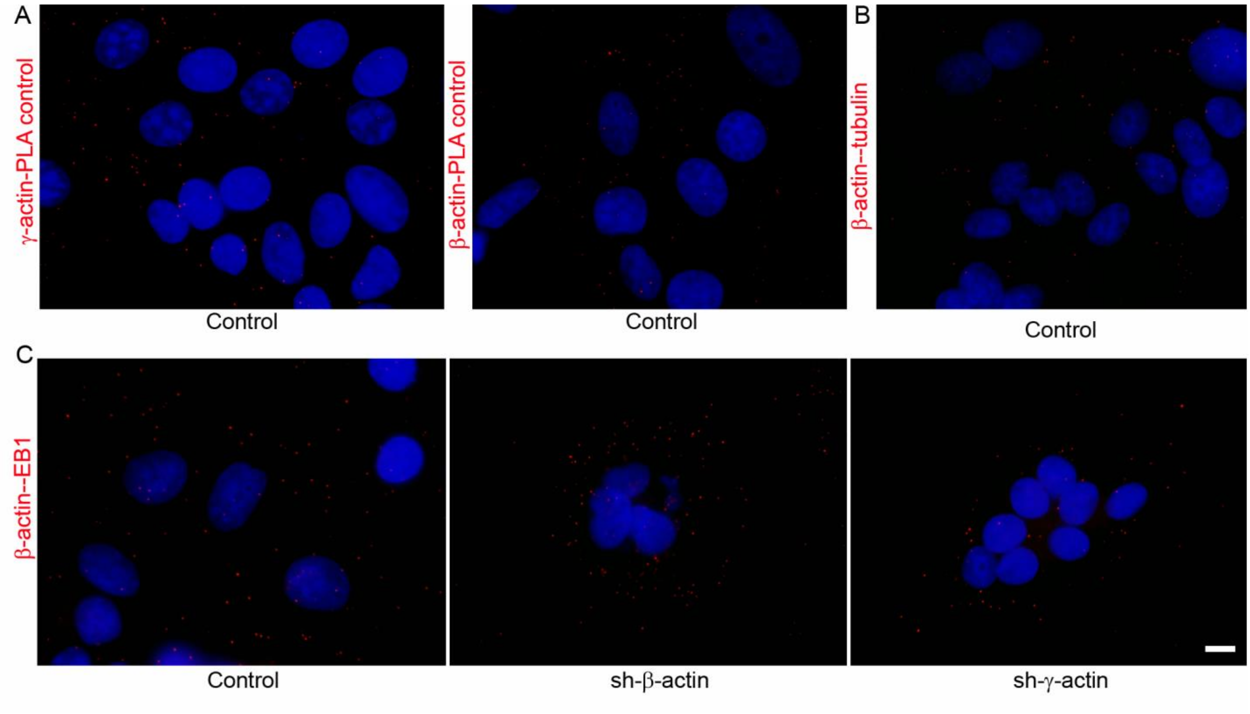

Supplementary Figure 4. Control PLA experiments.

A. PLA dots in γ-actin and β-actin antibody controls.

B. β-actin--tubulin PLA experiments.

C. β-actin--EB1 PLA experiments.

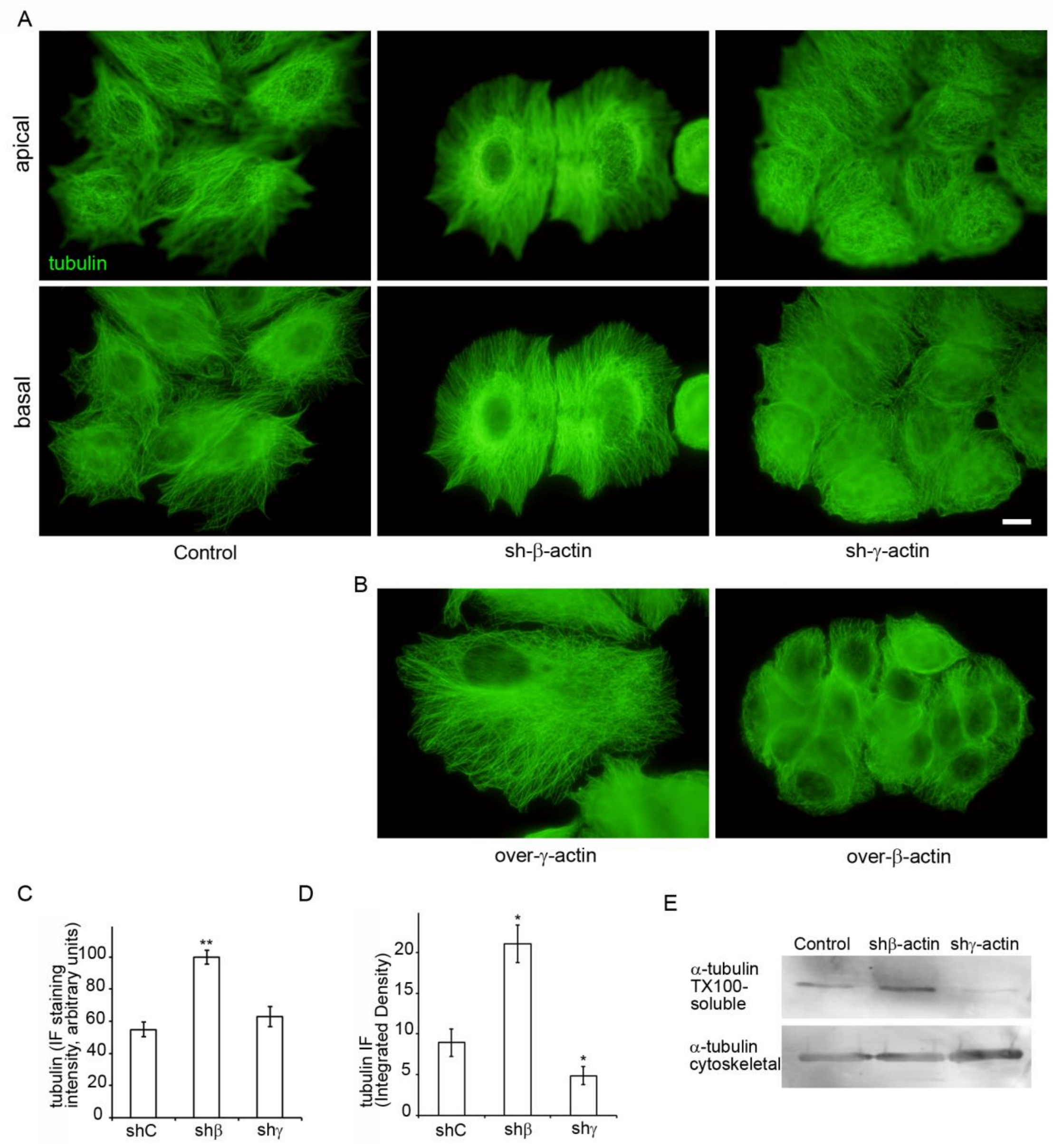

Supplementary Figure 5. Analysis of microtubule organization at the leading edge of MCF7 cells.

A. Microtubule organization at the basal and apical levels in MCF7 cells depleted of β- or γ-actin.

B. Microtubule organization at the basal level in MCF7 cells with exogenous expression of β- or γ-actin.

C. Microtubule fluorescence intensity and integrated density (D) at the leading edges of β- or γ-actin-depleted cells. (E) Tubulin polymerization levels after down-regulation of cytoplasmic actins in MCF7 cells. WB analysis. Data of a representative experiment.

A

B

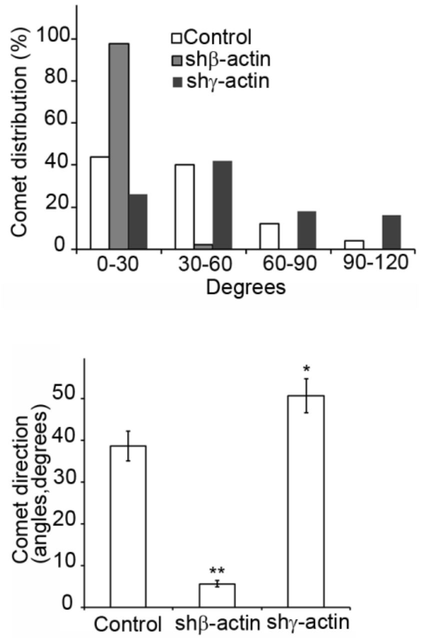

Supplementary Figure 6. Comparative analysis of microtubule directionality at the leading edge of MCF7 cells.

A. Histograms of the distribution of the angles between EB1 comets and radius-vectors at the growing microtubule ends of β- or γ-actin-depleted cells.

B. The average angles between EB1 comets and radius-vectors.
